# Validating Ultra-Wideband Positioning System for Precision Cow Tracking in a Commercial Free-Stall Barn

**DOI:** 10.3390/ani14223307

**Published:** 2024-11-17

**Authors:** Ágnes Moravcsíková, Zuzana Vyskočilová, Pavel Šustr, Jitka Bartošová

**Affiliations:** 1 Department of Ethology, Institute of Animal Science, 104 00 Praha, Czech Republic; moravcsikova.agnes@vuzv.cz (Á.M.); vyskocilova.zuzana@vuzv.cz (Z.V.);; 2 Department of Ethology and Companion Animal Science, Faculty of Agrobiology, Food and Natural Resources, Czech University of Life Sciences Prague, 165 00 Prague, Czech Republic

**Keywords:** UWB, positioning, dairy cow, indoor, accuracy, precision, proximity

## Abstract

UWB positioning systems offer innovative solutions for precision monitoring dairy cow behaviour and social dynamics, yet their performance in complex commercial barn environments requires thorough validation. This study evaluated the TrackLab 2.13 (Noldus) UWB system in a dairy barn housing 44–49 cows. We assessed stationary tag positioning using ten fixed tags over seven days, proximity detection between eight cows and ten stationary tags, and moving tag positioning using three tags on a stick to simulate cow movement. System performance varied by tag location, with reliability ranging from 4.09% to 96.73% and an overall mean accuracy of 0.126 ± 0.278 m for stationary tags. After the provider updated the software, only 0.62% of measures exceeded the declared accuracy of 0.30 m. Proximity detection between moving cows and stationary tags showed 81.42% accuracy within a 2-m range. While generally meeting specifications, spatial variations in accuracy and reliability were observed, particularly near barn perimeters. These findings highlight UWB technology’s potential for precision livestock farming, welfare assessment, and behaviour research, including social interactions and space use patterns. Results emphasise the need for careful system setup, regular updates, and context-aware data interpretation in commercial settings to maximise benefits in animal welfare monitoring.

## Simple Summary

Ultra-wideband (UWB) positioning systems offer promising solutions for monitoring dairy cow behaviour, but their performance in complex barn environments needs a thorough evaluation. This study assessed the TrackLab 2.13 UWB system (Noldus) in a commercial dairy barn, focusing on its accuracy and reliability for tracking cow movements and detecting proximities. We tested the system using stationary and moving tags that simulated cow movement. Positioning reliability varied widely for stationary tags, with central barn locations performing best. Stationary and moving tags satisfactorily complied with the manufacturer’s specified accuracy of 0.30 m. The system accurately detected 81.42% of cow proximities to fixed points, indicating its potential for studying social interactions and resource use. While challenges remain, particularly near barn perimeters, this UWB technology shows great promise for enhancing animal welfare monitoring and behaviour research in commercial dairy settings. These findings will help farmers and researchers utilise positioning systems more effectively, improving livestock management and welfare assessment.

## 1. Introduction

Positioning systems have become indispensable tools in modern dairy farming, addressing the livestock sector’s critical challenges. The global demand for animal products is projected to increase by 70% by 2050, necessitating significant improvements in livestock management [1]. This growth, coupled with the trend of increasing herd sizes and decreasing numbers of farmers, has created a scenario where more than traditional methods of animal monitoring are needed [2]. Various technological approaches have been developed for tracking and monitoring cattle behaviour. Traditional video-based systems using mounted cameras combined with computer vision can detect specific behaviours in cattle, as demonstrated, for example, by Porto et al. [3], who developed a system for automatically detecting the lying behaviour of dairy cows using multi-camera video recordings. Radio-frequency identification (RFID) systems offer reliable individual identification at specific points like feeding stations or milking parlours; however, they typically have limited spatial resolution, as reviewed by Ruiz-Garcia and Lunadei [4]. Radar-based positioning systems have also been employed, offering good accuracy but potentially being affected by metallic structures [5]. More recently, computer vision systems enhanced with deep learning have shown promise in identifying and tracking individual farm animals, though they require substantial training data and processing power [6].

The automated assessment of cattle welfare through technological advancements has the potential to decrease labour demands and enhance the reliability of evaluations by employing continuous, real-time monitoring [7]. Such automated monitoring systems provide farmers with comprehensive livestock data, which may increase production efficiency and improve animal welfare [8]. Simultaneously, livestock farming faces rising consumer concern about the conditions in which animals are raised, and food is produced [9,10]. Positioning systems can address these concerns by providing objective, continuous monitoring of spatial behaviour patterns. When combined with activity monitoring, these systems can detect behavioural changes associated with health issues, such as reduced feeding duration, indicating lameness [11]. Monitoring an animal’s spatial behaviour provides valuable information about its health, allowing farmers to intervene promptly and prevent animal suffering [12].

Such positioning technologies address industry challenges and revolutionise scientific research in animal behaviour. Positioning systems offer advantages over conventional methods, providing more efficient data collection than traditional direct observation or video analysis. For example, social interactions often need to be assessed over 24 h periods, including nighttime. Conventional methods would require additional lighting for nocturnal observations, potentially disrupting the animals’ circadian rhythms and influencing their behaviour. However, positioning systems allow continuous monitoring without such interventions, ensuring more natural behavioural data [5]. Monitoring cow activity using UWB technology and tracking circadian rhythms can aid in the early detection of health problems [13,14].

The system has been proven effective for detecting lameness through changes in spatial behaviour and activity patterns [11]. While behavioural changes associated with other health issues, such as mastitis, have been documented [15], the potential of UWB systems for detecting these conditions requires further research.

Additionally, it is feasible to observe the proximity interaction network within a herd [16], which may eventually allow for the detection of individual variations in social associations [17], potentially leading to the identification of health issues. Data obtained from UWB positioning systems can also be utilised to assess animal behaviours [18,19], including feeding habits [20] and oestrus behaviour [21,22].

For determining positions in complex indoor environments, UWB technology has become popular as a promising solution for animal tracking, offering high-precision positioning capabilities [23]. This technology is spatially and temporally even more accurate than Global Navigation Satellite Systems (GNSS) technology [24]. UWB-based systems typically comprise hardware elements (such as multiple anchors and tags) and software [25]. These systems transmit data at high rates over short distances with low power densities [26]. Short impulses are the most suitable for indoor environments like barns, as the high temporal resolution of UWB technology allows for precise tracking even in the presence of multiple reflections and refractions from metal structures. UWB technology can distinguish direct signals from reflected or refracted ones, which enhances accuracy in such environments [27,28]. As a result, these systems are more accurate in indoor environments—most UWB-based systems claim accuracy within centimetres, in contrast to technologies based on other principles, which typically report accuracy in metres [29].

Despite the potential of UWB technology, its application in commercial livestock settings presents unique challenges. The complex environment of a dairy barn, with its metal structures, varying animal densities, and specific microclimatic conditions, can affect the accuracy and reliability of positioning systems [30]. Missing data values from UWB devices might hinder reliable continuous monitoring and analysis of animal movement and social behaviour [31,32]. Therefore, rigorous validation of these systems in real-world settings is crucial to ensure their effectiveness for research and practical farm management applications [33].

While UWB positioning systems show promise for precision livestock monitoring, their real-world performance in commercial dairy barns needs to be validated, particularly regarding accuracy and reliability under challenging conditions such as metal structures and dynamic animal densities. Although several studies have tested UWB systems in controlled environments, more comprehensive validations are required in commercial dairy settings. In addition to static accuracy, proximity detection capabilities and performance with moving targets are critical aspects for practical applications in animal welfare monitoring and behavioural research.

This study has both scientific and applied aims in evaluating the performance of a positioning system based on UWB technology (the TrackLab 2.13, Noldus) in a commercial dairy barn environment. The scientific objective is to understand the capabilities and limitations of UWB technology in complex barn environments, particularly how physical structures and barn layout affect system performance. Specifically, we assessed the following:

1. The reliability and accuracy of stationary tags located in predefined places in the barn.
2. The accuracy of automatic proximity detection between moving cows and stationary tags.
3. The accuracy of moving tag positioning to simulate animal movement.

The practical aim is to provide insights for farmers and researchers into the real-world performance of UWB-based positioning system (PS) technology in commercial dairy settings, including potential challenges in different barn areas and factors affecting system accuracy when monitoring individual movement patterns and social interactions. This validation will help future users understand what to expect when implementing this technology for behavioural research and farm management purposes.

## 2. Materials and Methods

### 2.1. Experimental Barn and Animals 

**Figure 1:**
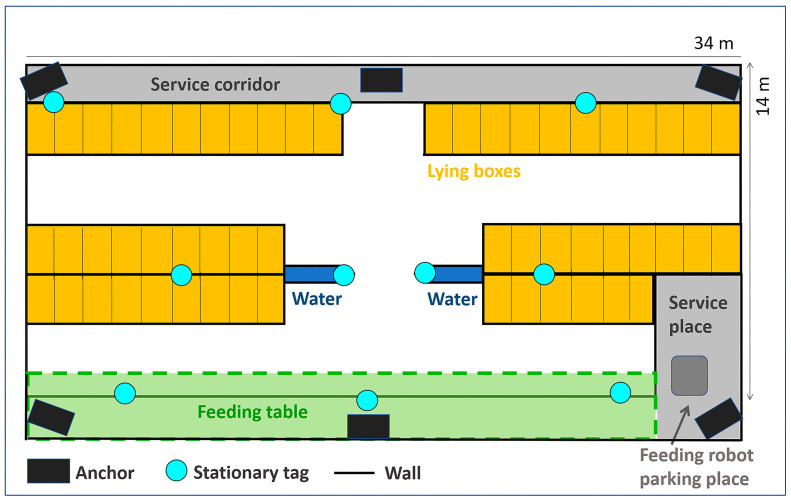
Scheme of the barn, including positions of stationary tags (blue circles) and anchors (black rectangles). Cows could freely move in the white area and enter the lying boxes (orange colour).

This study was conducted at the Institute of Animal Science’s experimental farm, Netluky, Prague, Czech Republic, from January 2023 to February 2023. A battery of tests was executed in a free-stall system barn for post-calving lactating cows, with an average age of 3.64 ± 1.37 years and an average of 2.33 ± 1.26 lactations. During the study, 44 to 49 Holstein Friesian and Czech Simental dairy cows were housed in a barn designed for 54 individuals. The interior space of the barn was divided into several functional zones: three rows of straw-bedded lying cubicles (54 in total, width 1.137 m, length 2.164 m, neck rail from the curb 1.066 m), centrally located drinking troughs, a feeding table, and corridors (see Figure 1). The barn management practices ensured good welfare conditions for the cows. The barn operated below its maximum capacity of 54 cows, allowing sufficient space for all individuals. Animals had continuous access to feed, water, and a mechanical rotating brush for grooming. The free-stall system enabled natural movement and social interactions among cows. Manure was removed from the alleys by a tractor twice daily during milking time when cows were out of the barn. The straw bedding in cubicles was fully replaced once a week, with a thin layer of fresh straw added daily to maintain comfortable lying conditions. The cows were fed a Total Mixed Ratio (TMR) based on corn haylage, hay, straw, and mineral additives. Fresh TMR was delivered twice daily and automatically pushed back to the feeding table when needed. A feeding robot was permanently stationed at the right end of the feeding table when not in operation. The cows were milked twice daily at 4–6 am and 3–5 pm in a tandem parlour with 5 × 5 stalls.

### 2.2. Ultra-Wideband Positioning System 

Positions in the barn were tracked using the TrackLab 2.13 (Noldus) positioning system (PS), based on UWB technology with a Real-Time Location System (RTLS) (Noldus Information Technology, Wageningen, Netherlands). The system consisted of 6 Sewio IP Sensors installed on the perimeter walls at the height of 4 m, covering the entire 31 m × 12 m area. The UWB tags (Sewio Leonardo, 54 mm × 40 mm × 14 mm, 26 g) were used to locate animals or fixed positions in the stable. The supplier company calibrated the system. The tag sampling frequency was set to 1 Hz, which means the system was configured to update the position information of each tag at one-second intervals. The PS uses a two-dimensional (X, Y) coordinate system to track tag location in the barn. The provider updated the system software during the study period (1 February 2023; regular online update). The update included reconfiguring the Z-coordinate settings in RTLS Manager, fixing it at 1.0 m to represent the average height of a tag when attached to a cow’s collar. The Z-coordinate was included in PS for future use in behavioural monitoring; the provider has not yet fully developed this functionality.

### 2.3. Validation of the Positioning System 

A set of tests was selected and adapted based on previously published studies to validate the PS in a commercial cattle barn and its future usability for automatically monitoring social distances in a group of cows. Tested tags were either (i) stationary, i.e., attached to the frame of the stable, to assess the success rates of positioning (‘reliability’) as well as the accuracy of positioning the exact location; (ii) moving, i.e., put on a real cow, to assess the ability of a PS to detect another tag in a certain distance; or (iii) fixed in a group of three on a stick that was moved throughout the barn by a person to check the accuracy of positioning the tags with a known short distance (0.3 m) that reflected accuracy announced by the supplier.

### 2.4. Reliability and Accuracy of Stationary Tag Positioning 

Ten tags (‘stationary tags’) were distributed at fixed locations throughout the stable, and their positioning (continuous measuring and sending the coordinates by PS) was run during a non-operated period between morning and evening milking for seven days between January 19 and February 6. This approach collected seven sequences lasting 120 to 275 min (24 h for each tag). Four observational days occurred before the provider updated the system, while three were after it.

The locations were chosen to represent areas that the cows often visited, and the signal could potentially be disturbed by barn equipment (e.g., metal constructions) or weakened due to their location (e.g., stable corners). Three stationary tags were placed in the feeding zone, three in the lying box zone near the end of the barn, two in the middle of the stable within the lying box area, and two in the middle of the barn near the water source (see Figure 1). The heights of installation ranged from 1 to 1.5 m, except for the tags in the feeding table area, which were placed at a height of about 2 m to keep them safe from animals. The stationary tags were removed when not used to prevent damage by the cows or a stable environment.

The success rates of obtaining positions of fixed tags (‘reliability’) were evaluated by calculating the ratio of actual numbers of position recordings to the expected number of recordings according to the given recording frequency of the system (one position per second).

To assess the accuracy of the fixed tag positions measured by PS, i.e., the range of distances between each and mean measure when positioning the same point, we first determined the centroid of the cluster of measured values for each observation. The cluster’s centroid was calculated as the arithmetic mean of all recorded positions within the observation period (day). Subsequently, using the Pythagorean theorem, we calculated the distance of each point measured by the system from the corresponding cluster centroid.

### 2.5. The Accuracy of Automatic Proximity Detection Between Moving and Stationary Tags 

Eight cows were randomly selected from the herd to detect the accuracy of the two tags meeting for a certain distance (‘proximity’) in the barn. The cows were equipped with collars bearing a tag and observed as focal individuals three times each continuously for one hour (i.e., three hours per cow). A total of 13 sessions occurred before and 11 after the provider updated the system. The tags stayed on the cows for the whole testing period. No health issues arose requiring the replacement of any of the monitored cows during the proximity detection testing. During the observation, the focal cow was followed by a trained observer (ZV) who marked her location in the barn in the protocol every 15 s. A threshold distance of 2 m, approximating the body length of a cow, was established for proximity detection. The task of a human observer was to record whether the focal cow (specifically, the tag on her neck) entered the 2-m range of any stationary tag. Essentially, each stationary tag had a defined circular zone with a 2-m radius around it. The exact time spent near the stationary tag was recorded once a cow entered the 2-m zone. In the case of positions recorded by PS, the Pythagorean theorem was applied to calculate the distance between the tag carried by the focal cow and each of the stationary tags for each recorded position. All distances shorter than 2 m were assessed as proximity. Concordance (YES/NO) between proximity determined by PS and the observer was assigned then. There were four possible scenarios: (1) congruence (no stationary tag or identical tag detected by both PS and observer); (2) incongruence (no stationary tag detected by PS while the person observed one); (3) incongruence (a stationary tag detected by PS while none by the observer); and (4) incongruence (the tag identity detected in the proximity of a cow differed).

### 2.6. Reliability and Accuracy of Moving Tag Positioning 

A dynamic motion test assessed the system’s accuracy in tracking moving targets. Three tags were attached to a stick, spacing them 0.3 m apart. An experimenter carried out the test by walking along a path that covered all the functional areas of the barn. It took 14 min. Throughout the test, the experimenter maintained a slow and steady pace to ensure consistent movement speed. The wooden stick with the attached tags was held horizontally approximately 1.5 m above the ground. The test was completed after the system update. The distances between the outer tags towards the middle one were calculated from the obtained coordinates according to the Pythagorean theorem when the positioning met the following criterion: the positioning occurred within the exact second.

### 2.7. Data Analysis 

We analysed the data using SAS for Windows version 9.4 (SAS Institute Inc., Cary, NC, USA). Descriptive statistics were conducted using the UNIVARIATE procedure. General linear models were employed to evaluate differences in the reliability and the accuracy of individual tags (PROC GLIMMIX) with the normal distribution and identity as link function. Within-group least squares means were calculated for classes of tested categorical variables (LSMEANS statement), i.e., tag’s ID and day of observation. The Tukey–Kramer adjustment was applied to correct multiple comparisons between means.

## 3. Results

The results are summarised in Table 1 and described in detail in the following chapters.

### 3.1. Reliability and Accuracy of Stationary Tag Positioning

**Figure 2:**
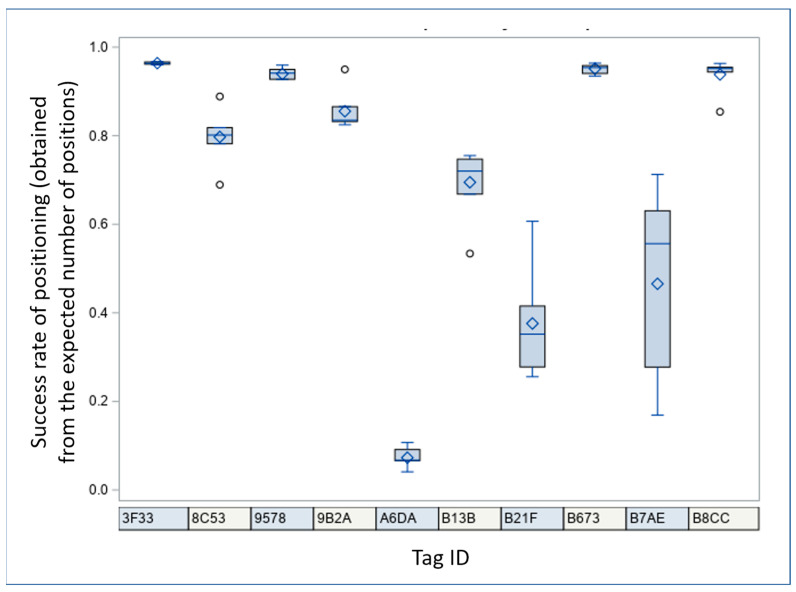
Reliability of stationary tag positioning over the observed period, i.e., the success rate obtained from the expected number of positions sent by the positioning system (raw data presented as side-by-side box plots, PROC UNIVARIATE, SAS). The central diamond sign in each box represents the sample mean. The centre horizontal line corresponds to the sample median, while the box boundaries to the interquartile range, the whiskers indicate the range, and the individual circles the outliers.

**Figure 3:**
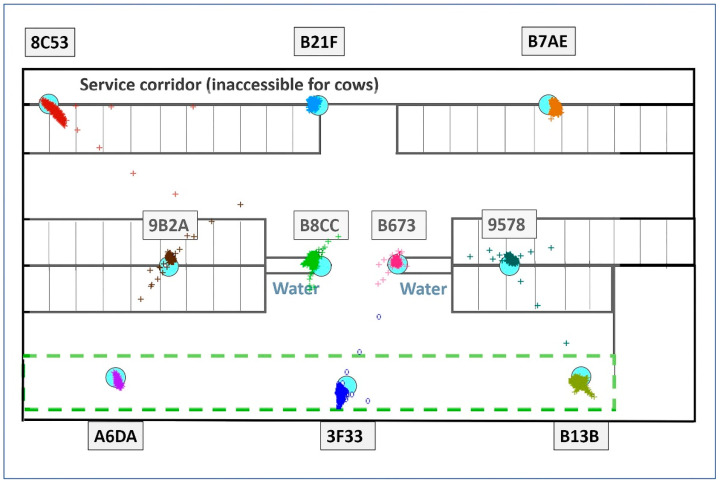
Positioning of the stationary tags after the provider updated the software. Blue circles indicate the position of the stationary tags in the barn, while clusters of differently coloured marks show the positions of each stationary tag measured by the positioning system.

The PS demonstrated considerable variability in the reliability of positioning the stationary tags fixed at different locations within the barn (see Figure 2). The reliability of positioning (the success rate of obtained positions from PS during an observing period) ranged from 4.09% to 96.73% across tags, with an overall mean of 70.55 ± 30.12% (mean ± standard deviation; expected number of recorded positions using every second positioning: 894,536.). It significantly differed among individual tags (F_(9, 54)_ = 88.23, *p* < 0.0001, general linear model, PROC GLIMMIX, SAS), but not on particular days (F_(6, 54)_ = 0.61, *p* = 0.72). The reliability did not change after the system update (F_(1, 59)_ = 0.01, *p* = 0.94). The location within the barn impacted the reliability of a tag. The tags placed in the corners exhibited the lowest reliability, especially A6DA in the lower left corner (see Figure 3). Contrastingly, the tags in the barn’s centre showed the highest reliability.

The mean time difference between two consecutive measures by PS (N = 629,504 differences) was 1.42 ± 2.47 s (median = 1.153 s; 95% shorter than 3.121 s; maximum = 437.71 s). Three tags were responsible for 97.6% of most prolonged delays (1%, more extended than 8.25 s) between consecutive measures: A6DA (54.3%), B21F (24.3%), and B7AE (19.0%).

Throughout the monitoring period, the precision (accuracy) of positioning (i.e., distance of a measure from the daily centroid) ranged from 0.0006 to 15.96 m, with an overall mean of 0.126 ± 0.278 m (N = 623,693 measures); 7.42% of the measures outreached the 0.3 m stated by the supplier. There were 0.56% measures larger than 2 m (the approximate length of a cow), 95.6% of which were accounted for by one tag (B13B). The overall means for particular tags ranged from 0.039 ± 0.038 m to 0.549 ± 0.686 m. Nine of the ten tags showed mean values that met the manufacturer’s specified accuracy of up to 0.3 m; 95% of measures were within this range in seven tags. The precision differences among the tags were confirmed by the statistical analysis (F_(9, 623,693)_ = 28,622.70, *p* < 0.0001). Contrary to the reliability assessments (see above), the date impacted accuracy (F_(6, 623,693)_ = 7229.88, *p* < 0.0001); the later (February) measures were significantly more precise than the earlier ones (January). The provider’s update of PS software was detected between those two periods. The precision considerably improved when analysing updated data collection separately. The overall mean of precision improved from 0.178 ± 0.323 m before the update (N = 341,165 measures) to 0.063 ± 0.066 m (N = 282,528 measures) after the update; only 0.62% of positioning exceeded the 0.3 m declared by the producer (see Figure 3). Still, there was individual variability among particular tags (F_(9, 282,528)_ = 6745.77, *p* < 0.0001). Nevertheless, all ten tags fulfilled the declared precision when reaching mean distances from the centroid between 0.035 ± 0.034 m (tag B673) and 0.119 ± 0.103 m (tag 8C53). The largest errors, again, were observed in the corners of the barn (tags 8C53, B13B, and A6DA), while the centrally located tags demonstrated the lowest errors (see Figure 3, graphs on the right).

### 3.2. The Accuracy of Automatic Proximity Detection Between Moving and Stationary Tags 

According to manual observation, focal cows (‘moving tags’) spent 39.5% of the total duration of 24 h near any stationary tag. The PS correctly identified the proximate stationary tag in 81.42% of the 409 cases where a focal cow appeared near a stationary tag. Two stationary tags placed in the upper corners (8C53 and B7AE) were responsible for most errors (7:56 from 9:00 min of proximity detection errors). All of these errors occurred before the provider updated the software.

Only twice, PS failed to detect a manually observed stationary tag (always generally problematic A6DA in the lower left corner); once, the wrong ID (4:24 min, before the system update); and once, no tag was detected despite the cow being observed close to a stationary tag (00:15 min, after the update). After the update, the proximity detection errors lasted 15 s from 11 h of observation.

Individual cows (i.e., moving tags on real cows) showed huge variability in positioning reliability, which was associated with animal-related influences; on average, variations from 22.2 to 76.9% were obtained from the expected measures. Within each 1 h observation, the reliability ranged from 2.5 to 89.9%. The average reliability after the system update was not significantly different from before the system update (50.0 ± 30.9 vs. 47.0 ± 23.1%).

### 3.3. Reliability and Accuracy of Moving Tag Positioning 

**Figure 4:**
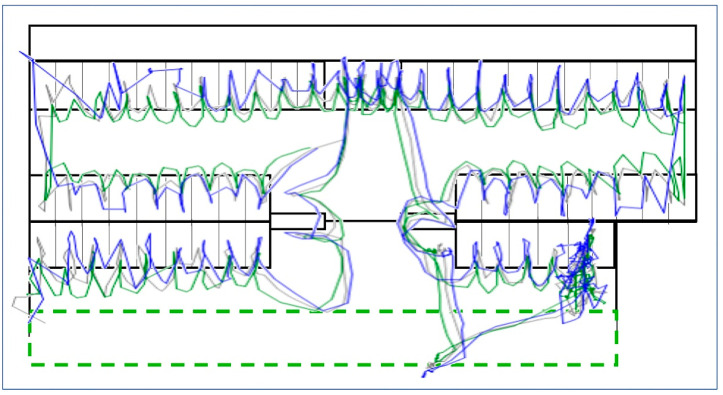
Reconstruction of the predefined path (14 min) through the barn during the dynamic motion test by the positioning system. An observer walked with three tags attached to a wooden stick at a fixed distance of 0.3 m. The trajectory was produced in SAS (PROC GPLOT, interpolation = join, no correction for missing values). Blue colour shows the track of the tag on the top of the stick, grey the middle one and green the tag closest to the handgrip.

The dynamic motion tests demonstrated the ability of PS to reconstruct the predefined path’s overall shape with varying deviation levels (Figure 4). Compared to 0.30 m of fixed distance on the stick, the mean distances between the central tag and each of the two peripheral ones across all measures were 0.41 ± 0.20 (tag 9578; range 0.01–1.59 m) and 0.42 ± 0.23 m (tag B7AE; range 0.01–1.78 m). Altogether, 84.6% of distances calculated from PS positioning met 0.30 m, as declared by PS’s producer. However, consistent with the stationary test results, more significant errors were observed when the tags approached the perimeter of the sensor network, particularly near the corners of the barn (see Figure 4).

The reliabilities of the three moving tags were comparable with well-operating stationary tags. The middle tag on the stick (grey in Figure 4) reached the best reliability, 87.7%, and the tag closest to the handgrip, 83.6% (tag 9578, green colour in Figure 4). The tag on the top of the stick (B7AE, the blue colour in Figure 4) obtained 73.4% measures from 855 possible ones.

## 4. Discussion

This study evaluated the performance of the UWB positioning system (TrackLab 2.13, Noldus) in a dairy barn environment, focusing on its accuracy and reliability across various positioning scenarios covering three critical aspects: (i) positioning of fixed locations, (ii) proximity detection between two tags, and (iii) moving tag positioning. PS performed enough to satisfy the performance declared by the supplier and predefined parameters for further use in tracking cows in the commercial barn. Its effectiveness and accuracy, however, considerably depended on tag location and movement within the barn. The challenges and limitations of UWB-based technology application in livestock environments and its implications for monitoring animal behaviour and welfare are further discussed.

### 4.1. Reliability and Accuracy of Stationary Tag Positioning 

The analysis of stationary tag positioning revealed significant spatial variability in both reliability and accuracy within the barn environment. This variability has important implications for deploying and using UWB systems in dairy settings.

Positioning reliability: The success rates for obtaining valid positions ranged widely (from 4.09% to 96.73%). It indicates that tag location critically influences system reliability. Tags placed in the centre of the barn, including the middle part of a feeding table, consistently showed the highest reliability. At the same time, those in corner locations, particularly in the lower left corner, exhibited the poorest reliability. For example, issues with the B13B tag before the software update may have been associated with its proximity to a parked feeding robot. The metal construction of the robot could have caused electromagnetic interference or signal reflection, which may have adversely affected its performance.

This spatial variation aligns with findings from previous studies on UWB systems in complex indoor environments. For example, Melzer et al. [27] reported excellent performance (>99% detection rates) of the UWB RTLS (Ubisense) for most stationary tags, but noted one tag with a significantly lower detection rate of about 12% in a challenging position. Location-based performance associated with the challenging environment has also been reported in other technologies, e.g., in a radar-based local positioning measurement (LPM) system. For example, Gygax et al. [5] observed an increased rate of missing estimates in areas near walls and metal structures. In our case, the lower left corner had the most physical barriers (a wall dividing the barn from a small area with a brush and a gate through which the feed wagon passed). These factors and the general challenges of corner locations likely explain why this area showed the poorest performance in our positioning system.

Also, the calibration quality can significantly affect system performance, potentially interacting with the effects of tag placement. For example, Melzer et al. [27] markedly improved detection rates for cow-mounted tags after switching from laser calibration to professional surveying calibration (from 66.85–67.38% to 79.37–80.38% of expected measurements). In our study, the supplier calibrated the UWB system immediately after installation and before validation. Later, additional calibration was performed during the validation due to regular software updates, which further increased the positioning precision. Thus, keeping the system updated according to the development made by the supplier can yield further benefits.

Our results and experience strongly support the suggestion made by Melzer et al. [27] to leave reference tags in the barn. When placed at known locations throughout the barn, these reference tags allow for detecting any drift or degradation in system performance. Additionally, they can help identify if specific anchors are underperforming, which allows for timely maintenance or recalibration of the system. Researchers can significantly improve the overall quality of positioning data in barn environments by combining regular software updates, proper calibration, and reference tags.

Time between consecutive measures: The analysis of time intervals between consecutive measures revealed the variable performance of the UWB system. The mean time difference of 1.42 ± 2.47 s (median 1.153 s) indicated certain data collection inconsistencies that the system’s specifications may not capture. This variability in positioning intervals is a critical consideration for researchers and practitioners, particularly when studying behaviours that occur over short time scales. Detailed examination of measurement intervals represents a new approach to evaluating positioning system performance. Many studies have described system performance by reporting measurement intervals between consecutive position estimates (e.g., 1 s [34] and 1.73 s [27]). Our research added insights into the distribution of positioning failures throughout the measurement. This approach could be valuable for future studies as it provides a more accurate picture of system reliability and consistency.

Accuracy of position measurements: The positioning system demonstrated high precision overall, with a mean distance from the daily centroid of 0.126 ± 0.278 m. This level of precision is suitable for many applications in animal behaviour studies, particularly for larger animals like cattle. Our results are comparable with other UWB systems reported in the literature. For instance, Zhuang et al. [28], using a Ubisense UWB system, reported an overall accuracy of 0.37 m at 1 m height and 0.50 m at 0.3 m height. Porto et al. [34], also validating a Ubisense UWB positioning system, achieved slightly better results with their reference tag, reporting a mean error of about 0.11 m. However, we observed significant spatial variations in precision (0.0006 to 15.96 m), with more significant errors occurring in the corners of the feeding area, while centrally located tags showed lower errors. These findings align, for example, with D’Urso et al. [35], who validated a Sewio UWB system and reported that accuracy for points measured on the perimeter was significantly lower than that for points measured in the centre of the barn. After the actualisation of the software by the provider, all ten tags fulfilled the declared precision of 0.3 m, with mean distances from the centroid ranging between 0.035 ± 0.034 m and 0.119 ± 0.103 m. The largest errors were again observed in the corners of the feeding area, while the centrally located tags demonstrated the lowest errors.

This marked improvement in precision following the provider’s software update underscores the critical role of ongoing system maintenance and updates in animal tracking studies. The findings highlight the importance of researchers staying informed about and implementing the latest software versions to ensure data quality and precision in their research.

Different filtering and smoothing methods have been commonly used to address measurement inaccuracies in positioning systems. Three main types of errors can be identified in UWB positioning data: significant reflection errors characterised by unrealistic jumps of 2–15 m between samples, normal random variations in position, and missing data [20]. Signal loss often occurs in areas near metal tubes, plywood walls, and concrete posts or when signals are blocked by other animals [30]. Each error type requires a specific approach—jump filters can eliminate reflection errors, while median filtering [30] or Kalman filtering [27] can smooth out random variations. Interpolation methods can handle missing data [20]. However, as Melzer et al. [27] demonstrated, these filtering methods might not be necessary when the RTLS system is accurately calibrated. This finding emphasises the importance of proper system calibration and regular updates over post-processing corrections.

### 4.2. The Accuracy of Automatic Proximity Detection Between Moving and Stationary Tags 

Positioning systems in farmed animals are increasingly used to study social relationships and group dynamics (e.g., [36,37]), moving beyond traditional applications of tracking individual locations and behaviours. In addition, we focused on validating the accuracy of proximity detection by the UWB system (TrackLab 2.13, Noldus). This may be invaluable for automatically assessing interindividual distances and social relationships among group members that human observers cannot reach. We found a reasonable level of accuracy in proximity detection between tags carried by cows and stationary tags. Proximity detection errors represented 0.625% of the 24 h observation period. PS correctly identified 81.33% of cases when a cow approached the stationary tag for less than 2 m (the approximate length of a cow). The high accuracy rate is particularly noteworthy given the dynamic nature of a commercial dairy environment. This accuracy and the low error rate suggest the system can reliably track cow movements and interactions with specific points of interest (e.g., scratching brushes, water trough, another cow). This is crucial for studies focusing on resource use patterns, such as feeding and drinking behaviours and social interactions between cows.

Comparisons between studies of this kind are always challenging due to the methodological differences. Still, our results can be contextualised within the broader literature on proximity detection via indoor positioning systems. For instance, Tullo et al. [38], validating the CowView UWB PS, found high sensitivity and precision for feeding detection (0.97 and 0.94, respectively) and cubicle occupancy (0.95 and 0.98), while standing in the alley showed lower sensitivity (0.63) but still good precision (0.87). These results demonstrate the system’s capability to accurately detect critical behaviours based on the cow’s location in predefined zones of the barn. Another example was Melzer et al. [27], who compared two approaches (zone- and distance-based approach) for determining neighbour preferences with a Ubisense UWB. At the lying stalls, the zone approach provided the lowest error and high correlation with video observations. After accurate system calibration, the zone approach performed best for the feed bunk.

Our approach, focusing on validating proximity detection between cows and stationary tags, provides flexibility and adapts easily to various barn layouts without relying on predefined zones. It offers higher spatial resolution, allowing a more detailed analysis of cow movements and interactions. Combining proximity-based and zone-based approaches may provide a more comprehensive understanding of cow behaviour and social interactions. This hybrid approach could leverage the strengths of both methods: zone-based systems excel at identifying broad behavioural patterns, while proximity-based systems offer detailed insights into specific interactions. This level of detail could be valuable for studying social dynamics, feeding hierarchies, and individual preferences within the herd. While our study focused on validating cow-to-stationary tag proximity detection accuracy, it opens up new possibilities for more nuanced livestock monitoring systems.

### 4.3. Reliability and Accuracy of Moving Tag Positioning 

Previous studies have utilised predefined paths and directional changes to assess the accuracy and responsiveness of positioning systems under realistic movement patterns. For example, Zhuang et al. [28] validated the UWB tracking system by simulating complex sow movement. This approach is instrumental in ensuring that the system reliably captures rapid shifts in position, as commonly observed in livestock. Such trajectory tests help identify potential limitations in accurately tracking movement, especially in dynamic and unpredictable farm conditions.

Our dynamic tag test demonstrated the UWB system’s capability to track moving targets accurately. Figure 4 visualises how PS reconstructed the path that a person accomplished throughout the barn that aimed to simulate the movement of a cow, i.e., walking along the solid equipment of the barn and visiting every single lying box with a stick equipped with three tags with fixed distances. During this on-farm test, i.e., when a person could not keep the stick horizontally and not tilted all the time, 84.6% of distances calculated from measured coordinates fell within the 0.30 m range declared by the supplier. This performance indicates the system’s potential for capturing the complex movement patterns of dairy cows. However, the increased errors observed near the barn’s perimeter echo the limitations in the stationary tests, highlighting a consistent challenge for UWB systems in livestock environments.

Our results align with findings from similar studies using UWB technology. For example, in a pig barn, Zhuang et al. [28] achieved 0.38 m accuracy while tracking moving tags at 1 m height with an Ubisense UWB, while Hindermann et al. [24] reported deviations less than 0.5 m for most measurements at withers height, measured by DecaWave UWB. The latter study observed consistent patterns across tags, including location-specific errors, likely due to barn equipment interference. These findings corroborate our observations on the impact of the barn environment on UWB accuracy, as discussed in detail in the 4.1 chapter.

### 4.4. Limitations of the Study 

While this study provides valuable insights into the performance of the TrackLab 2.13 (Noldus) UWB positioning system in a dairy barn environment, it is important to acknowledge several limitations that inform the interpretation of our results and highlight areas for future research.

Firstly, our study was conducted in a single commercial dairy barn. This barn’s specific layout, construction materials, and environmental conditions may only be representative of some dairy facilities. Future research should validate these findings across various barn designs and environments to ensure generalizability. Secondly, our observations were conducted over a relatively short period. Long-term studies are needed to assess the system’s reliability over extended periods and under varying environmental conditions (e.g., seasonal changes and different stocking densities). Additionally, we did not explore the impact of herd size on system performance. As commercial dairy operations can vary significantly in size, understanding how the system performs under different stocking densities is crucial for its practical application.

The study also revealed the significant impact of software updates on system accuracy. While this highlights the importance of keeping the system up to date, it also raises questions about the comparability of data collected before and after such updates. However, updates, such as those made during our experiment and other setting adjustments, are a standard part of installing and optimising UWB systems. Therefore, as a practical recommendation to other system users, we suggest not avoiding updates to ensure the most accurate information.

Researchers should be aware of this potential source of variation in longitudinal studies. Lastly, due to methodological differences, direct comparisons with other studies in the field were challenging. This limitation underscores the need for standardised testing protocols to validate livestock positioning systems.

Despite these limitations, our study provides a solid foundation for understanding the capabilities and challenges of UWB positioning systems in commercial dairy settings. Future research addressing these limitations will be crucial for this technology’s continued refinement and application in precision livestock farming and animal behaviour research.

## 5. Conclusions

In conclusion, the UWB positioning system (the TrackLab 2.13, Noldus) demonstrates promising capabilities for tracking dairy cow movements and detecting proximities in a commercial barn setting. However, its performance is influenced by spatial factors within the barn, highlighting the need for careful system setup and data interpretation. 

Our validation shows that farmers and researchers can use this technology to conduct the following:

- Track individual cow locations throughout the barn with validated accuracy levels;
- Monitor how often and how long cows visit specific barn areas;
- Detect proximities between cows and critical resources (e.g., feeding areas, water sources) and proximity to another cow;
- Record movement patterns across different functional areas of the barn.

As UWB technology evolves, addressing the identified limitations and expanding on the strengths revealed in this study will be crucial for its successful application in precision livestock farming and animal behaviour research. Future studies should focus on long-term system reliability under varying environmental conditions and performance validation with different herd sizes and barn layouts.

## Figures and Tables

**Table 1:** Summary of the main results.

Reliability and Accuracy of Stationary Tags	
Range of Reliability	4.1–96.7%
Mean Reliability ± Standard Deviation	70.6% ± 30.1%
Impact of the System Update on Reliability	No
Range of Accuracy (Distances from Centroid)	0.0006–15.9 m
Impact of System Update on Accuracy	Yes (*p* < 0.0001)
Accuracy before Update	0.18 ± 0.32 m
Accuracy after Update	0.063 ± 0.066 m
Proximity Detection between Moving and Stationary Tags (Tags on Cows)	
Correct Proximity Detection by the PS	81.4% of 409 Cases of Observed Proximity
Impact of System Update on Proximity Detection	Yes (all failures occurred before the Update)
Reliability of Moving Tags on Individual Cows	22.2–76.9%
Impact of the System Update on Reliability	No
Reliability and Accuracy of Moving Tags (on Stick)	
Reliability of Moving Tags	Middle tag: 87.7% Tag near Grip: 83.6% Tag on the Top: 73.4%
Calculated Distance to Middle Tag on Stick (Real Distance = 0.30 m)	Tag near Grip: 0.41 ± 0.20 m Tag on the Top: 0.42 ± 0.23 m

## Data Availability

According to a general policy of the Institute of Animal Science, the data are available upon request. Please contact the corresponding author if you are interested.
